# Flow Status-Based Predicted Prosthesis-Patient Mismatch in Patients Undergoing Transcatheter Aortic Valve Replacement With a Balloon-Expandable Valve

**DOI:** 10.1016/j.shj.2024.100379

**Published:** 2024-12-06

**Authors:** Daijiro Tomii, Dik Heg, Masaaki Nakase, Daryoush Samim, Jonas Lanz, Fabien Praz, Stefan Stortecky, David Reineke, Stephan Windecker, Thomas Pilgrim

**Affiliations:** a Department of Cardiology, Bern University Hospital, Inselspital, University of Bern, Bern, Switzerland; b Department of Clinical Research, University of Bern, Bern, Switzerland; c Department of Cardiovascular Surgery, Bern University Hospital, Inselspital, University of Bern, Bern, Switzerland

**Keywords:** Aortic stenosis, Effective orifice area, Flow status, Prosthesis-patient mismatch, Transcatheter aortic valve replacement

## Abstract

**Background:**

Effective orifice area (EOA) is flow dependent. However, established methods for the assessment of predicted prosthesis-patient mismatch (PPM) do not consider flow status and therefore may underestimate the rate and impact of PPM in patients with abnormal flow status. This study aimed to investigate the clinical impact of flow status-based predicted PPM in patients undergoing transcatheter aortic valve replacement (TAVR).

**Methods:**

Patients undergoing TAVR in a prospective TAVR registry were stratified by the presence of moderate or severe PPM (EOA index to body surface area [EOAi]: 0.65-0.85 or ≤0.65 and 0.55-0.70 or ≤0.55 cm^2^/m^2^ if obese). PPM was defined according to echocardiographically measured EOAi (measured PPM) or predicted or flow status-based predicted EOAi. Predicted EOAs were based on reference values of EOA for each valve generation and size (predicted PPM_THV_) or native aortic annulus dimension (predicted PPM_CT_).

**Results:**

Among 1510 patients included (August 2007-June 2022), rates of moderate or severe PPM differed according to method of assessment: 27.0 and 8.7% according to measured PPM, 11.3 and 1.2% according to predicted PPM_THV_, 12.0 and 0.1% according to PPM_CT_, 21.6 and 0.2% according to flow status-based predicted PPM_THV_, and 25.1 and 0.4% according to flow status-based predicted PPM_CT_. Five-year mortality was comparable in patients with and without flow status-based predicted PPM defined by either method. These results were consistent when patients were stratified by flow status.

**Conclusions:**

Rates of PPM differ considerably when flow status is considered. There was no consistent signal of increased risk of adverse events up to 5 years in patients with flow status-based predicted PPM.

**Clinical Trial Registration:**

https://www.clinicaltrials.gov. NCT01368250.

## Highlights

• In the assessment of prosthesis-patient mismatch (PPM) after transcatheter aortic valve replacement, the use of predicted vs. measured effective orifice area results in a lower estimate of PPM frequency.
• Consideration of flow status significantly recategorized the severity of PPM.
• Flow status-based predicted PPM was not associated with an increased risk of adverse events up to 5 years of follow-up irrespective of flow status.
• Further studies are warranted to optimize accurate assessment of bioprosthetic hemodynamics and to evaluate the impact of PPM on long-term clinical outcomes after transcatheter aortic valve replacement.

## Introduction

Prosthesis-patient mismatch (PPM) is a condition of nonstructural bioprosthetic valve dysfunction in which the effective orifice area (EOA) of a normally functioning prosthesis is small relative to the patient's body surface area (BSA), resulting in an increased transprosthetic pressure gradient. In surgical series, PPM is an established predictor of adverse clinical outcomes.^1^, ^2^, ^3^ In transcatheter aortic valve replacement [TAVR] populations, the clinical impact of PPM remains controversial due to differences in the methods used to determine PPM.^4^ Historically, PPM in the TAVR population has been defined using EOA measured directly by transthoracic echocardiography (measured PPM).^5^^,^^6^ Recent evidence suggests that predicted EOA, calculated by dividing the reference EOA based on prosthesis model and size of the native aortic annulus dimension, defines PPM more specifically compared to measured EOA and therefore may be more useful in assessing its impact on clinical outcomes.^7^, ^8^, ^9^, ^10^ However, standard predicted EOA does not take flow variability into account and may therefore distort PPM assessment in patients with reduced flow. Recently, Akinmolayemi and colleagues proposed new reference values of predicted EOA according to flow status (flow status-based EOA) based on data from the PARTNER trials and registry.^11^ The clinical relevance of PPM based on flow status-based EOA (flowstatus-based predicted PPM) has not been evaluated so far. The present study aimed to systematically evaluate the frequency and clinical impact of flow status-based predicted PPM in a prospective TAVR registry.

## Methods

### Study Design and Population

The Bern TAVI registry, part of the nationwide SwissTAVI registry (registered at clinicaltrials.gov with NCT01368250),^12^ is a prospective TAVR registry enrolling consecutive patients undergoing TAVR for severe, symptomatic aortic stenosis at Bern University Hospital, Switzerland. The present analysis included patients who underwent TAVR with balloon-expandable devices (SAPIEN XT, SAPIEN 3, SAPIEN 3 Ultra [Edwards Lifesciences, Irvine, California]) between August 2007 and June 2022. For the purpose of the present study, patients who underwent intervention but had no device implanted, who were treated with nonstudy devices, or patients with incomplete information for the assessment of PPM (body mass index [BMI], BSA, measured EOA, or stroke volume [SV] indexed to BSA [SVI] at discharge) were excluded. The registry was approved by the Bern ethics committee, and patients provided written informed consent to participate.

### Definition of PPM

PPM was classified as no, moderate, or severe PPM on the basis of EOA indexed to BSA (EOAi) according to the Valve Academic Research Consortium (VARC)-3 definitions. For patients with BMI <30 kg/m^2^, no PPM was defined as EOAi >0.85 cm^2^/m^2^, moderate PPM as EOAi >0.65 and ​≤ ​0.85 cm^2^/m^2^, and severe PPM as EOAi ≤0.65 cm^2^/m^2^. For patients with BMI ≥30 kg/m^2^, no PPM was defined as EOAi >0.70 cm^2^/m^2^, moderate as EOAi >0.55 and ​≤ ​0.70 cm^2^/m^2^, and severe PPM as EOAi ≤0.55 cm^2^/m^2^.^13^

### Assessment of EOA

Measured and predicted EOA were assessed as previously described.^10^ Measured EOA was calculated by the continuity equation using the left ventricular stroke volume, derived as the outer-to-outer border of the stented valve, multiplied by the pulsed-wave Doppler time-velocity integral of flow at that location.^14^^,^^15^ Predicted EOA was based on the reference values of EOA indicated by the published data for each size and generation of balloon-expandable valve implanted (predicted EOA_THV_) or on the reference values of EOA derived from aortic annulus dimension measured by preprocedural computed tomography (CT) (predicted EOA_CT_). The reference values for predicted EOA were derived from published data, which were calculated using data from pooled cohorts of the randomized clinical trials.^7^, ^8^, ^9^, ^10^ For the assessment of flow status-based predicted EOA, patients were stratified by post-TAVR flow status. Flow status was determined by SVI at discharge (low flow; SVI <35 mL/m^2^ and normal flow; SVI ≥35 mL/m^2^).^16^ The published normal reference values of EOA for each balloon-expandable device size and generation (flow status-based predicted EOA_THV_) or native aortic annulus area (flow status-based predicted EOA_CT_) were then applied (Supplementary Tables 1 and 2).^11^ The reference values of predicted and flow status-based predicted EOA_CT_ were used in patients who underwent TAVR with SAPIEN 3/3 Ultra. Preprocedural CT examinations and post-TAVR echocardiography were independently re-evaluated by dedicated imaging specialists, and the measurements were integrated into the database.^17^^,^^18^

### Data Collection and Clinical Endpoints

All baseline clinical, procedural, and follow-up data were prospectively recorded in a dedicated database held at the Clinical Trials Unit at the University of Bern, Switzerland. In the SwissTAVI registry, regular follow-up is standardized at 30 days and at 1, 5, and 10 years.^10^ Clinical follow-up data were obtained by standardized interviews, documentation from referring physicians, and hospital discharge summaries. All adverse events were systematically collected and adjudicated by an independent clinical events committee based on the VARC definitions.^19^^,^^20^ An independent analyst at the Clinical Trials Unit is responsible for central data monitoring to verify the completeness and accuracy of the data and for statistical analysis. The outcomes of interest in the present study included all-cause and cardiovascular mortality, structural valve deterioration, and unplanned repeat aortic valve intervention at 1 and 5 years after TAVR. Structural valve deterioration was defined according to the VARC criteria between 2007 and 2013 and has since been defined according to the VARC-2 criteria.^10^^,^^19^^,^^20^ Unplanned repeat aortic valve intervention was defined as a composite of valve-in-valve procedure, balloon valvuloplasty, surgical revision, and paravalvular leak closure.

### Statistical Analysis

Categorical variables are presented as frequencies and percentages, and the differences between groups were evaluated with the chi-square test or Fisher exact test. Continuous variables are presented as mean values ​± ​SD and compared between groups using Student’s t-test. Risk ratios with 95% CIs from Poisson regressions were provided where appropriate. Cumulative incidence curves were constructed using the Kaplan-Meier method. Cox proportional hazards models were used to calculate hazard ratios and 95% CIs for the clinical outcomes. Multivariable adjustment was performed with predefined baseline variables potentially related to clinical outcomes including age, sex, and the Society of Thoracic Surgeons Predicted Risk of Mortality. The Fine and Gray method was used to model the cumulative incidence function of the outcomes of interest in the present study and to determine the subdistribution hazard ratio under competing risk with death, or in the case of cardiovascular death under competing risk with noncardiovascular death.^21^^,^^22^ All statistical tests were two-sided, and *p*-values of <0.05 were considered significant. Statistical analyses were performed using Stata 17 (StataCorp, College Station, TX).

## Results

### Study Population and Frequency of PPM

**Figure 1:**
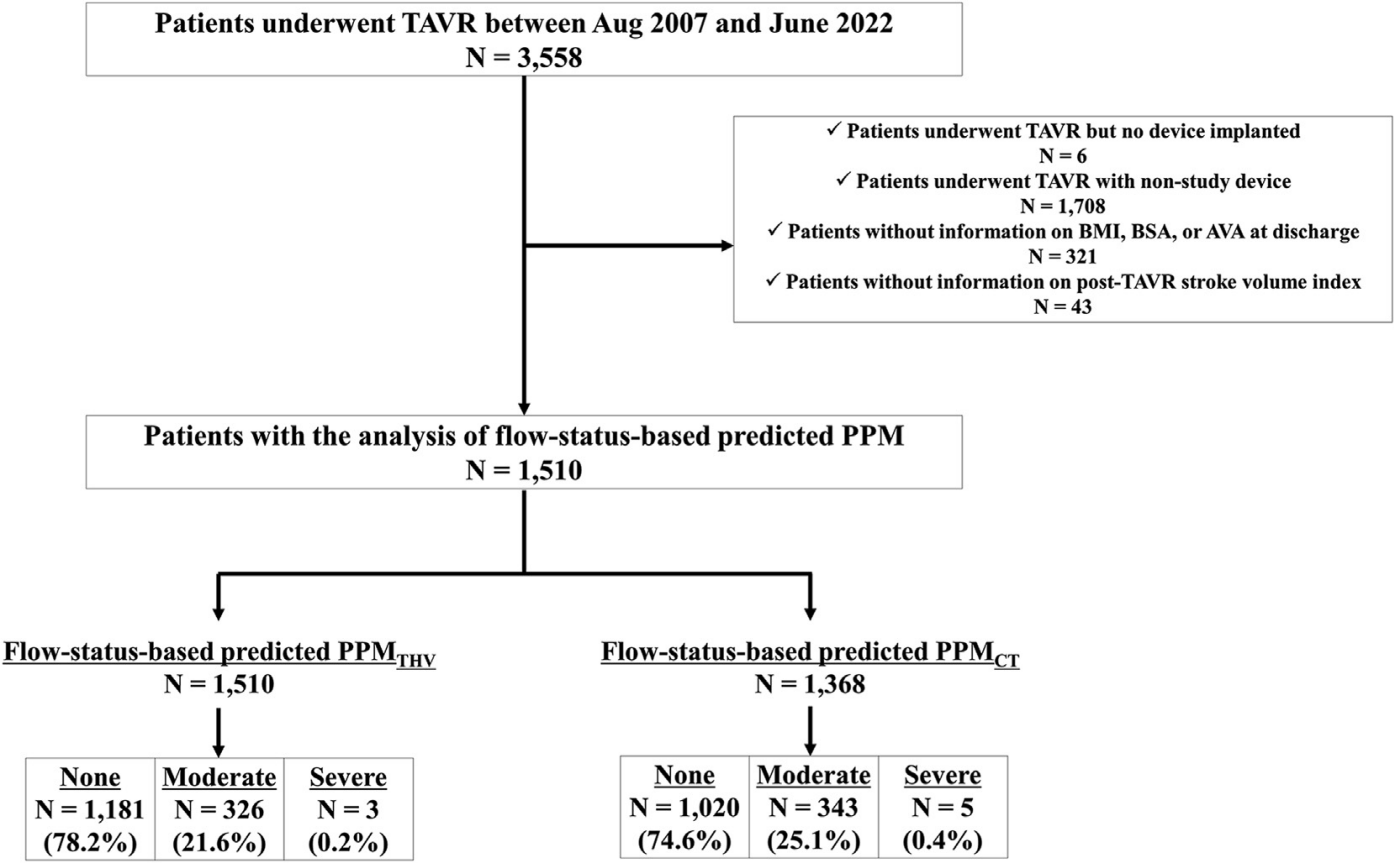
Study flowchart. Abbreviations: AVA, ​aortic valve area; BMI, ​body mass index; BSA, ​body surface area; PPM, ​prosthesis-patient mismatch; PPM_CT_, PPM defined by the normal reference values of effective orifice area derived from aortic annulus area/perimeter measured by preprocedural computed tomography; PPM_THV_, PPM defined by the normal reference values of effective orifice area for each size and model of implanted transcatheter heart valve; TAVR, ​transcatheter aortic valve replacement.

**Figure 2:**
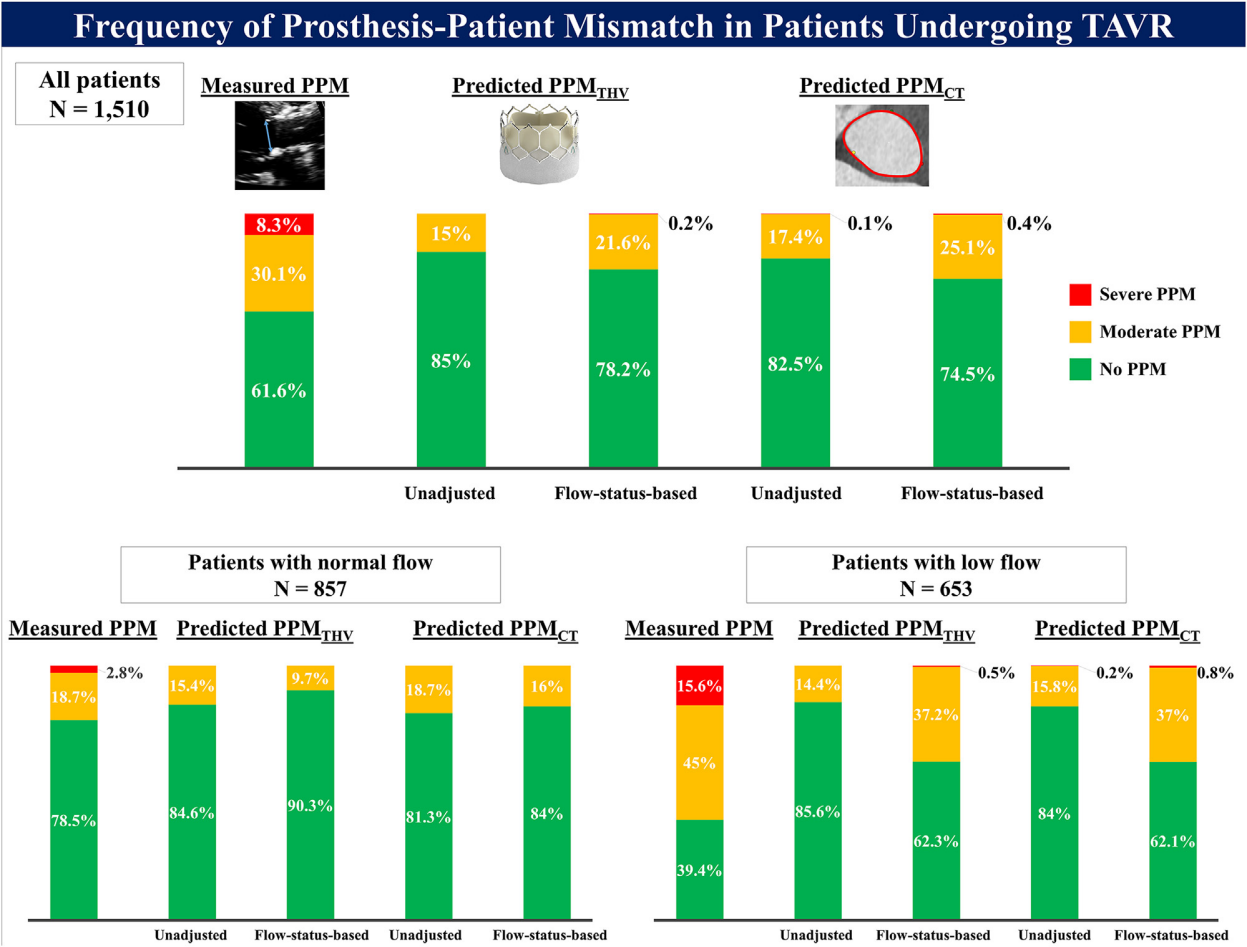
Frequency of prosthesis-patient mismatch in patients undergoing TAVR frequency of PPM according to type and severity after TAVR. Abbreviations: PPM, ​prosthesis-patient mismatch; PPM_CT_, PPM defined by the normal reference values of effective orifice area derived from aortic annulus area/perimeter measured by preprocedural computed tomography; PPM_THV_, PPM defined by the normal reference values of effective orifice area for each size and model of implanted transcatheter heart valve; TAVR, ​transcatheter aortic valve replacement.

Among 3588 patients enrolled in the institutional TAVR registry, 1510 and 1368 patients were included in the analysis of flow status-based predicted PPM defined by flow status-based predicted EOA_THV_ (PPM_THV_) and flow status-based predicted PPM defined by flow status-based predicted EOA_CT_ (PPM_CT_), respectively (Figure 1). Normal and low flow were present in 857 (56.8%) and 653 (43.2%) patients, respectively, at discharge after TAVR. The frequencies of moderate and severe PPM defined by measured EOA were 30.1 and 8.3% in the overall population, 18.7 and 2.8% in patients with normal flow, and 45 and 15.6% in patients with low flow. The frequencies of moderate and severe PPM were lower using predicted EOA_THV_ (15 and 0% in the overall population, 15.4 and 0% in patients with normal flow, and 14.4 and 0% in patients with low flow) and predicted EOA_CT_ (17.4 and 0.1% in the overall population, 18.7 and 0% in patients with normal flow, and 15.8 and 0.2% in patients with low flow). When flow status-based predicted EOA was applied, the frequency of moderate or severe predicted PPM substantially increased in patients with low flow status (37.2 and 0.5% for predicted PPM_THV_ and 37 and 0.8% for predicted PPM_CT_), whereas the frequencies decreased in patients with normal flow (9.7 and 0% for predicted PPM_THV_ and 16 and 0% for predicted PPM_CT_) (Table 1 and Figure 2).

**Table 1:** Frequency of PPM according to the method for definition of PPM

	All patients (N ​= ​1510)	Patients with normal flow (N ​= ​857)	Patients with low flow (N ​= ​653)
Measured PPM			
Measured EOAi (cm^2^/m^2^)	0.93 ​± ​0.28	1.04 ​± ​0.29	0.79 ​± ​0.20
Moderate or severe measured PPM, n (%)	580 (38.4%)	184 (21.5%)	396 (60.6%)
Moderate measured PPM, n (%)	454 (30.1%)	160 (18.7%)	294 (45.0%)
Severe measured PPM, n (%)	126 (8.3%)	24 (2.8%)	102 (15.6%)
Predicted PPM_THV_			
Predicted EOAi_THV_ (cm^2^/m^2^)	0.92 ​± ​0.12	0.92 ​± ​0.12	0.91 ​± ​0.12
Moderate or severe predicted PPM_THV_, n (%)	226 (15.0%)	132 (15.4%)	94 (14.4%)
Moderate predicted PPM_THV_, n (%)	226 (15.0%)	132 (15.4%)	94 (14.4%)
Severe predicted PPM_THV_, n (%)	0	0	0
Flow status-based predicted PPM_THV_			
Flow status-based predicted EOAi_THV_ (cm^2^/m^2^)	0.91 ​± ​0.14	0.97 ​± ​0.13	0.84 ​± ​0.11
Moderate or severe flow status-based predicted PPM_THV_, n (%)	329 (21.8%)	83 (9.7%)	246 (37.7%)
Moderate flow status-based predicted PPM_THV_, n (%)	326 (21.6%)	83 (9.7%)	243 (37.2%)
Severe flow status-based predicted PPM_THV_, n (%)	3 (0.2%)	0	3 (0.5%)
Predicted PPM_CT_	N ​= ​1368	N ​= ​779	N ​= ​589
Predicted EOAi_CT_ (cm^2^/m^2^)	0.91 ​± ​0.12	0.92 ​± ​0.12	0.90 ​± ​0.12
Moderate or severe predicted PPM_CT_, n (%)	240 (17.5%)	146 (18.7%)	94 (16.0%)
Moderate predicted PPM_CT_, n (%)	239 (17.5%)	146 (18.7%)	93 (15.8%)
Severe predicted PPM_CT_, n (%)	1 (0.1%)	0	1 (0.2%)
Flow status-based predicted PPM_CT_			
Flow status-based predicted EOAi_CT_ (cm^2^/m^2^)	0.90 ​± ​0.14	0.95 ​± ​0.14	0.84 ​± ​0.11
Moderate or severe flow status-based predicted PPM_CT_, n (%)	348 (25.4%)	125 (16.0%)	223 (37.9%)
Moderate flow status-based predicted PPM_CT_, n (%)	343 (25.1%)	125 (16.0%)	218 (37.0%)
Severe flow status-based predicted PPM_CT_, n (%)	5 (0.4%)	0	5 (0.8%)

*Notes.* Values are mean ​± ​SD or n (%).

Abbreviations: EOAi, ​effective orifice area index to body surface area; EOAi_CT_, effective orifice area derived from preprocedural computed tomography index to body surface area; EOAi_THV_, effective orifice area for each size and model of implanted transcatheter heart valve index to body surface area; PPM, ​prosthesis-patient mismatch; PPM_CT_, PPM defined by predicted effective orifice area derived from preprocedural computed tomography; PPM_THV_, PPM defined by predicted effective orifice area for each size and model of implanted transcatheter heart valve.

Baseline and procedural characteristics according to flow status-based predicted PPM are shown in Table 2. TAVR was performed by transfemoral access in more than 90% of patients with no difference between groups. Patients with flow status-based predicted PPM were younger and had a lower prevalence of BMI ≥30 kg/m^2^, despite a higher mean BMI overall. Indexed aortic valve area was smaller in patients with flow status-based predicted PPM than in those without PPM. Patients with moderate or severe flow status-based predicted PPM were more likely to have a transcatheter heart valve (THV) size ≤23 mm compared with those without PPM. These results were consistent when patients were stratified into normal and low flow status after TAVR (Supplementary Tables 3 and 4).

**Table 2:** Baseline and procedural characteristics according to flow status-based predicted PPM

	Flow status-based predicted PPM_THV_			Flow status-based predicted PPM_CT_		
	No PPM (N ​= ​1181)	Moderate or severe PPM (N ​= ​329)	*p*-value	No PPM (N ​= ​1020)	Moderate or severe PPM (N ​= ​348)	*p*-value
Age, y	81.5 ​± ​6.5	80.6 ​± ​7.0	0.028	81.5 ​± ​6.5	80.5 ​± ​6.7	0.009
Female, n (%)	433 (36.7%)	129 (39.2%)	0.402	378 (37.1%)	130 (37.4%)	0.949
Body mass index, kg/m^2^	26.5 ​± ​5.0	28.7 ​± ​5.4	<0.001	26.4 ​± ​5.0	28.7 ​± ​5.4	<0.001
Body mass index ≥30 kg/m^2^, n (%)	308 (26.1%)	62 (18.8%)	0.007	265 (26.0%)	69 (19.8%)	0.021
Body surface area, m^2^	1.9 ​± ​0.2	2.0 ​± ​0.2	<0.001	1.9 ​± ​0.2	2.0 ​± ​0.2	<0.001
STS-PROM, %	4.7 ​± ​3.6	4.5 ​± ​3.8	0.372	4.6 ​± ​3.5	4.2 ​± ​3.6	0.086
NYHA III or IV, n (%)	689 (58.4%)	205 (62.3%)	0.205	603 (59.2%)	195 (56.0%)	0.314
TAVR for degenerative prosthesis, n (%)	22 (1.9%)	9 (2.7%)	0.377	15 (1.5%)	7 (2.0%)	0.466
Concomitant diseases						
Hypertension, n (%)	1033 (87.5%)	300 (91.2%)	0.066	888 (87.1%)	319 (91.7%)	0.021
Diabetes mellitus, n (%)	322 (27.3%)	105 (31.9%)	0.111	267 (26.2%)	108 (31.0%)	0.082
Renal failure (eGFR <60 mL/min/1.73 m^2^), n (%)	740 (62.7%)	174 (52.9%)	0.001	630 (61.8%)	172 (49.4%)	<0.001
Coronary artery disease, n (%)	711 (60.2%)	185 (56.2%)	0.205	613 (60.1%)	198 (56.9%)	0.312
Previous history						
Atrial fibrillation, n (%)	375 (31.8%)	118 (35.9%)	0.163	321 (31.5%)	118 (33.9%)	0.425
Peripheral artery disease, n (%)	140 (11.9%)	44 (13.4%)	0.447	120 (11.8%)	43 (12.4%)	0.774
Baseline echocardiography						
Indexed aortic valve area, cm^2^/m^2^	0.28 ​± ​0.09	0.25 ​± ​0.08	<0.001	0.28 ​± ​0.08	0.27 ​± ​0.08	0.050
Mean aortic valve gradient, mmHg	39.5 ​± ​15.7	39.1 ​± ​14.9	0.693	39.7 ​± ​15.8	39.5 ​± ​15.1	0.822
Left ventricular ejection fraction, %	55.2 ​± ​13.5	54.4 ​± ​14.1	0.373	54.8 ​± ​13.9	56.8 ​± ​12.0	0.017
Moderate or severe aortic regurgitation, n (%)	100 (8.5%)	32 (9.8%)	0.507	86 (8.4%)	33 (9.5%)	0.581
Moderate or severe mitral regurgitation, n (%)	172 (16.8%)	57 (20.9%)	0.129	147 (16.9%)	44 (14.8%)	0.467
Moderate or severe tricuspid regurgitation, n (%)	100 (10.4%)	35 (14.5%)	0.087	82 (10.0%)	34 (12.5%)	0.258
Procedural characteristics						
General anesthesia, n (%)	204 (17.3%)	67 (20.4%)	0.195	169 (16.6%)	61 (17.5%)	0.679
Femoral main access site, n (%)	1082 (91.6%)	303 (92.1%)	0.822	943 (92.5%)	324 (93.1%)	0.812
Valve size, mm	26.4 ​± ​2.1	24.8 ​± ​1.9	<0.001	26.5 ​± ​2.1	25.0 ​± ​1.9	<0.001
Valve size ≤23 mm, n (%)	204 (17.3%)	150 (45.6%)	<0.001	174 (17.1%)	147 (42.2%)	<0.001
Predilations, n (%)	679 (57.5%)	175 (53.4%)	0.186	572 (56.1%)	172 (49.4%)	0.034
Postdilations, n (%)	149 (12.6%)	54 (16.4%)	0.082	135 (13.2%)	52 (14.9%)	0.417

*Notes.* Values are mean ​± ​SD or n (%).

Abbreviations: eGFR, ​estimated glomerular filtration rate; NYHA, New York Heart Association; PPM, prosthesis-patient mismatch; PPM_CT_, PPM defined by predicted effective orifice area derived from preprocedural computed tomography; PPM_THV_, PPM defined by predicted effective orifice area for each size and model of implanted transcatheter heart valve; STS-PROM, ​Society of Thoracic Surgeons Predicted Risk of Mortality; TAVR, ​transcatheter aortic valve replacement.

### Postprocedural Hemodynamics

Postprocedural hemodynamics according to flow status-based predicted PPM are summarized in Table 3. Mean prosthetic gradients were higher in patients with moderate or severe flow status-based predicted PPM, defined by either method, compared with those without PPM under normal flow and low flow status. Patients with moderate or severe flow status-based predicted PPM had a higher prevalence of an increased transprosthetic gradient as compared to those without PPM in normal flow status, whereas the prevalence was comparable between the 2 groups in patients with low flow. Measured EOAi was lower in patients with severe or moderate flow status-based predicted PPM compared to those with no PPM under normal flow and low flow status. There was no significant difference in the rate of moderate or severe paravalvular leak.

**Table 3:** Post-TAVR valve hemodynamics

	Flow status-based predicted PPM_THV_					
	Normal flow			Low flow		
	No PPM (N ​= ​774)	Moderate or severe PPM (N ​= ​83)	*p*-value	No PPM (N ​= ​407)	Moderate or severe PPM (N ​= ​246)	*p*-value
Stroke volume index at discharge, ml/m^2^	45.8 ​± ​9.8	43.4 ​± ​7.3	0.031	28.4 ​± ​4.6	28.1 ​± ​5.1	0.413
Post-mean gradient, mmHg	11.8 ​± ​4.1	15.4 ​± ​5.4	<0.001	9.6 ​± ​4.0	11.0 ​± ​4.0	<0.001
Mean gradient ≥20 mmHg, n (%)	28 (3.6%)	19 (23.5%)	<0.001	10 (2.5%)	7 (2.9%)	0.802
Measured EOAi, cm^2^/m^2^	1.05 ​± ​0.3	0.88 ​± ​0.23	<0.001	0.82 ​± ​0.20	0.75 ​± ​0.20	<0.001
Moderate or severe aortic leak, n (%)	14 (1.8%)	2 (2.5%)	0.658	10 (2.5%)	9 (3.7%)	0.471

	Flow status-based predicted PPM_CT_					
	Normal flow			Low flow		
	No PPM (N ​= ​654)	Moderate or severe PPM (N ​= ​125)	*p*-value	No PPM (N ​= ​366)	Moderate or severe PPM (N ​= ​223)	*p*-value
Stroke volume index at discharge, ml/m^2^	45.5 ​± ​9.5	45.3 ​± ​9.5	0.855	28.4 ​± ​4.7	28.1 ​± ​5.0	0.401
Post-mean gradient, mmHg	11.7 ​± ​4.0	14.4 ​± ​5.5	<0.001	9.6 ​± ​3.7	11.0 ​± ​4.1	<0.001
Mean gradient ≥20 mmHg, n (%)	23 (3.5%)	21 (17.1%)	<0.001	6 (1.6%)	9 (4.1%)	0.102
Measured EOAi, cm^2^/m^2^	1.05 ​± ​0.28	0.94 ​± ​0.26	<0.001	0.81 ​± ​0.19	0.75 ​± ​0.20	0.001
Moderate or severe aortic leak, n (%)	12 (1.9%)	3 (2.5%)	0.717	12 (3.3%)	5 (2.3%)	0.614

Abbreviations: EOAi, effective orifice area index to body surface area; PPM, ​prosthesis-patient mismatch; PPM_CT_, PPM defined by predicted effective orifice area derived from preprocedural computed tomography; PPM_THV_, PPM defined by predicted effective orifice area for each size and model of implanted transcatheter heart valve; TAVR, ​transcatheter aortic valve replacement.

### Clinical Outcomes

**Figure 3:**
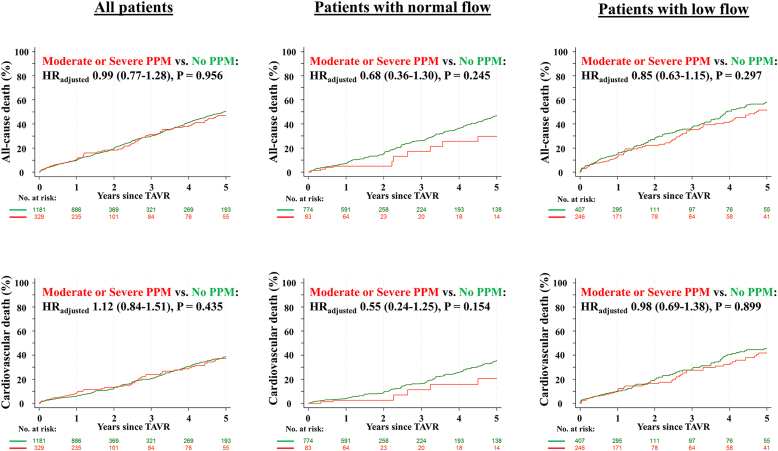
**Cumulative event curves for clinical outcomes according to flow status-based predicted PPM**_**THV**_**All-cause (upper) and cardiovascular death (lower) according to flow status-based predicted PPM**_**THV**_. Adjusted hazard ratios and *p*-values were calculated with the use of Cox proportional hazards models. Abbreviations: HR, ​hazard ratio; PPM, ​prosthesis-patient mismatch; PPM_THV_, PPM defined by the normal reference values of effective orifice area for each size and model of implanted transcatheter heart valve; TAVR, ​transcatheter aortic valve replacement.

During a median follow-up of 405 (interquartile range: 364; 1770) days, 446 patients died after TAVR up to 5 years after the procedure. Table 4 shows clinical outcomes according to flow status-based predicted PPM_THV_. In the overall population, all-cause death at 1 and 5 years occurred in 9.8 and 50.5% of patients with no PPM, and in 10.3 and 46.9% of patients with moderate or severe flow status-based predicted PPM_THV_ (Figure 3). There was no significant difference in all-cause or cardiovascular mortality between groups. Rates of structural valve deterioration, repeat aortic valve intervention, and persistent heart failure symptoms (New York Heart Association III/IV) at 5 years were comparable between groups. These results were consistent when patients were stratified by flow status.

**Table 4:** Clinical outcomes according to flow status-based predicted PPM

	Flow status-based predicted PPM_THV_											
	Entire cohort				Normal flow				Low flow			
	No PPM (N ​= ​1181)	Moderate or severe PPM (N ​= ​329)	HR_adjusted_ (95% CI)	Adjusted *p*-value	No PPM (N ​= ​774)	Moderate or severe PPM (N ​= ​83)	HR_adjusted_ (95% CI)	Adjusted *p*-value	No PPM (N ​= ​407)	Moderate or severe PPM (N ​= ​246)	HR_adjusted_ (95% CI)	Adjusted *p*-value
At 1 y												
All-cause death, n (%)	114 (9.8)	33 (10.3)	1.08 (0.73-1.60)	0.687	54 (7.1)	4 (4.9)	0.83 (0.30-2.34)	0.731	60 (14.9)	29 (12.2)	0.82 (0.52-1.28)	0.378
Cardiovascular death, n (%)	67 (5.8)	26 (8.3)	1.47 (0.93-2.31)	0.101	29 (3.8)	2 (2.5)	0.84 (0.20-3.60)	0.813	38 (9.6)	24 (10.3)	1.09 (0.65-1.82)	0.744
Repeat aortic valve intervention, n (%)	9 (0.8)	4 (1.3)	1.51 (0.46-4.91)	0.496	5 (0.7)	2 (2.4)	3.12 (0.58-16.79)	0.185	4 (1.0)	2 (0.9)	0.71 (0.13-3.97)	0.698
Structural valve deterioration, n (%)	23 (2.1)	7 (2.3)	1.09 (0.47-2.54)	0.846	17 (2.3)	2 (2.5)	1.12 (0.25-4.96)	0.880	6 (1.7)	5 (2.2)	1.27 (0.38-4.21)	0.696
NYHA III or IV, n (%)	96/1029 (9.3)	21/280 (7.5)	0.81 (0.52-1.27)	0.363	60/693 (8.7)	7/78 (9.0)	1.01 (0.47-2.16)	0.982	36/336 (10.7)	14/202 (6.9)	0.66 (0.36-1.19)	0.164
At 5 y												
All-cause death, n (%)	301 (50.5)	78 (46.9)	0.99 (0.77-1.28)	0.956	175 (46.8)	10 (29.7)	0.68 (0.36-1.30)	0.245	126 (57.8)	68 (51.4)	0.85 (0.63-1.15)	0.297
Cardiovascular death, n (%)	200 (38.7)	58 (37.1)	1.12 (0.84-1.51)	0.435	114 (35.3)	6 (20.5)	0.64 (0.28-1.46)	0.287	86 (45.2)	52 (41.7)	0.98 (0.69-1.38)	0.899
Repeat aortic valve intervention, n (%)	11 (1.5)	7 (5.0)	2.10 (0.81-5.45)	0.129	7 (1.6)	2 (2.4)	2.41 (0.49-11.98)	0.281	4 (1.0)	5 (5.8)	1.49 (0.38-5.88)	0.566
Structural valve deterioration, n (%)	29 (3.9)	14 (11.0)	1.66 (0.87-3.16)	0.122	21 (4.0)	3 (6.7)	1.49 (0.43-5.13)	0.524	8 (3.9)	11 (12.6)	1.76 (0.69-4.49)	0.237
NYHA III or IV, n (%)	38/270 (14.1)	14/73 (19.2)	1.33 (0.76-2.32)	0.312	29/191 (15.2)	4/20 (20.0)	1.26 (0.51-3.17)	0.616	9/79 (11.4)	10/53 (18.9)	1.55 (0.65-3.72)	0.324

	Flow status-based predicted PPM_CT_											
	Entire cohort				Entire cohort				Entire cohort			
	None (N ​= ​1020)	Moderate or severe PPM (N ​= ​348)	HR_adjusted_ (95% CI)	Adjusted *p*-value	None (N ​= ​654)	Moderate or severe PPM (N ​= ​125)	HR_adjusted_ (95% CI)	Adjusted *p*-value	None (N ​= ​366)	Moderate or severe PPM (N ​= ​223)	HR_adjusted_ (95% CI)	Adjusted *p*-value
At 1 y												
All-cause death, n (%)	97 (9.6)	29 (8.5)	0.91 (0.60-1.39)	0.675	39 (6.0)	7 (5.6)	1.12 (0.50-2.54)	0.781	58 (16.0)	22 (10.2)	0.63 (0.39-1.04)	0.070
Cardiovascular death, n (%)	59 (5.9)	22 (6.6)	1.15 (0.70-1.89)	0.573	20 (3.1)	4 (3.3)	1.28 (0.43-3.83)	0.658	39 (11.0)	18 (8.4)	0.78 (0.44-1.38)	0.395
Repeat aortic valve intervention, n (%)	8 (0.8)	5 (1.5)	1.72 (0.56-5.27)	0.345	6 (0.9)	1 (0.8)	0.75 (0.09-6.31)	0.790	2 (0.6)	4 (1.9)	2.78 (0.50-15.60)	0.245
Structural valve deterioration, n (%)	23 (2.4)	7 (2.1)	0.87 (0.37-2.04)	0.753	17 (2.7)	2 (1.6)	0.61 (0.14-2.66)	0.511	6 (1.9)	5 (2.4)	1.19 (0.36-3.98)	0.779
NYHA III or IV, n (%)	78/891 (8.8)	22/305 (7.2)	0.84 (0.53-1.33)	0.462	52/591 (8.8)	8/117 (6.8)	0.78 (0.38-1.59)	0.491	26/300 (8.7)	14/188 (7.4)	0.88 (0.46-1.67)	0.688
At 5 y												
All-cause death, n (%)	235 (47.6)	71 (46.9)	1.05 (0.80-1.37)	0.740	126 (43.5)	18 (38.1)	1.01 (0.61-1.67)	0.957	109 (54.9)	53 (51.1)	0.85 (0.61-1.19)	0.353
Cardiovascular death, n (%)	151 (34.7)	54 (38.8)	1.27 (0.93-1.74)	0.133	77 (30.9)	13 (32.1)	1.21 (0.66-2.20)	0.536	74 (41.6)	41 (42.1)	1.01 (0.69-1.49)	0.957
Repeat aortic valve intervention, n (%)	10 (1.7)	6 (2.9)	1.71 (0.62-4.72)	0.303	7 (1.7)	1 (0.8)	0.66 (0.08-5.38)	0.694	3 (1.9)	5 (4.0)	2.43 (0.56-10.45)	0.235
Structural valve deterioration, n (%)	26 (3.6)	11 (7.2)	1.25 (0.61-2.54)	0.541	19 (3.8)	4 (8.0)	1.16 (0.39-3.44)	0.791	7 (3.3)	7 (7.1)	1.51 (0.52-4.37)	0.452
NYHA III or IV, n (%)	35/227 (15.4)	11/68 (16.2)	1.05 (0.56-1.96)	0.889	27/158 (17.1)	3/26 (11.5)	0.69 (0.23-2.08)	0.508	8/69 (11.6)	8/42 (19.0)	1.56 (0.59-4.16)	0.371

*Notes.* Only the first outcome of each outcome type is counted (% from Kaplan-Meier estimates). HRs from Cox's regression (HR with 95% CIs) were also adjusted for age, gender, and STS-PROM score (adjusted HR with 95% CIs). Adjusted ratio ratios for NYHA (number of patients with NYHA III or IV/number of patients with NYHA assessed).

Abbreviations: HR, ​hazard ratio; PPM, ​prosthesis-patient mismatch; PPM_CT_, PPM defined by predicted effective orifice area derived from preprocedural computed tomography; PPM_THV_, PPM defined by predicted effective orifice area for each size and model of implanted transcatheter heart valve; NYHA, New York Heart Association; STS-PROM, ​Society of Thoracic Surgeons Predicted Risk of Mortality.

**Figure 4:**
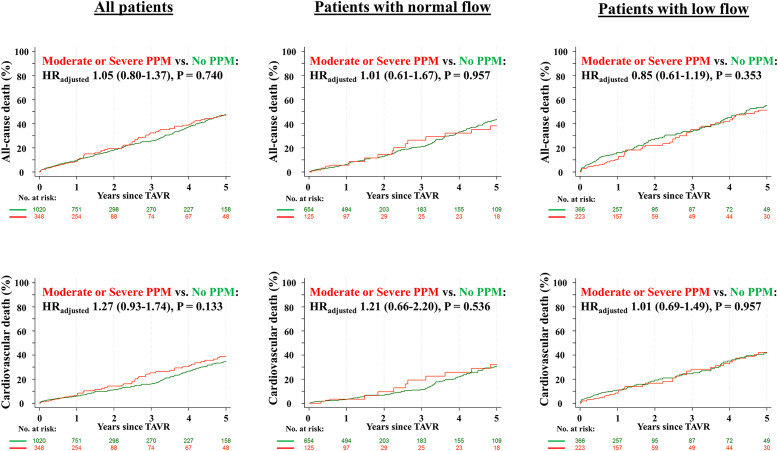
**Cumulative event curves for clinical outcomes according to flow status-based predicted PPM**_**CT**_**all-cause (upper) and cardiovascular death (lower) according to flow status-based predicted PPM**_**THV**_. Adjusted hazard ratios and *p*-values were calculated with the use of Cox proportional hazards models. Abbreviations: HR, ​hazard ratio; PPM, ​prosthesis-patient mismatch; PPM_CT_, PPM defined by the normal reference values of effective orifice area derived from aortic annulus area/perimeter measured by preprocedural computed tomography; PPM_THV_, PPM defined by the normal reference values of effective orifice area for each size and model of implanted transcatheter heart valve; TAVR, ​transcatheter aortic valve replacement.

Clinical outcomes at 1 and 5 years according to flow status-based predicted PPM_CT_ are summarized in Table 4. All-cause death at 1 and 5 years occurred in 9.6 and 47.6% of patients with no PPM, and in 8.5 and 46.9% of patients with moderate or severe flow status-based predicted PPM_CT_, respectively (Figure 4). There were no significant differences in all-cause or cardiovascular mortality and the rates of structural valve deterioration, repeat aortic valve intervention, and persistent heart failure symptoms between groups, regardless of flow status.

### Competing Risk Analysis

The results of the competing risk survival analysis for the outcomes are shown in Table 5. Consistent with the main analysis, moderate or severe flow status-based predicted PPM was not associated with an increased risk of cardiovascular death, structural valve deterioration, and repeat aortic valve intervention, regardless of the EOA definition and flow status.

**Table 5:** Clinical outcomes according to flow status-based predicted PPM in a competing risk analysis

	Flow status-based predicted PPM_THV_											
	Entire cohort				Normal flow				Low flow			
	None (N ​= ​1181)	Moderate or severe PPM (N ​= ​329)	HR_adjusted_ (95% CI)	Adjusted *p*-value	None (N ​= ​774)	Moderate or severe PPM (N ​= ​83)	HR_adjusted_ (95% CI)	Adjusted *p*-value	None (N ​= ​407)	Moderate or severe PPM (N ​= ​246)	HR_adjusted_ (95% CI)	Adjusted *p*-value
At 1 y												
Cardiovascular death, n (%)	67 (5.7)	26 (8.1%)	1.46 (0.90-2.37)	0.126	29 (3.8)	2 (2.4)	0.83 (0.20-3.50)	0.801	38 (9.4)	24 (10.0)	1.09 (0.64-1.86)	0.754
Repeat aortic valve intervention, n (%)	9 (0.8)	4 (1.2)	1.51 (0.47-4.88)	0.491	5 (0.7)	2 (2.4)	3.13 (0.62-15.70)	0.166	4 (1.0)	2 (0.8)	0.73 (0.13-4.09)	0.721
Structural valve deterioration, n (%)	23 (2.0)	7 (2.2)	1.09 (0.47-2.53)	0.840	17 (2.2)	2 (2.5)	1.12 (0.24-5.22)	0.883	6 (1.5)	5 (2.1)	1.36 (0.40-4.65)	0.622
At 5 y												
Cardiovascular death, n (%)	200 (34.1)	58 (36.3)	1.11 (0.82-1.52)	0.500	114 (31.6)	6 (19.4)	0.65 (0.29-1.48)	0.304	86 (40.1)	52 (38.7)	0.99 (0.69-1.41)	0.947
Repeat aortic valve intervention, n (%)	11 (1.4)	7 (3.1)	2.12 (0.82-5.45)	0.120	7 (1.2)	2 (3.5)	2.47 (0.52-11.65)	0.254	4 (1.5)	5 (3.1)	1.65 (0.44-6.20)	0.460
Structural valve deterioration, n (%)	29 (3.7)	14 (6.4)	1.68 (0.90-3.13)	0.102	21 (3.6)	3 (5.2)	1.51 (0.43-5.32)	0.525	8 (3.3)	11 (7.3)	1.98 (0.78-5.04)	0.153

	Flow status-based predicted PPM_CT_											
	Entire cohort				Normal flow				Low flow			
	None (N ​= ​1020)	Moderate or severe PPM (N ​= ​348)	sHR_adjusted_ (95% CI)	Adjusted *p*-value	None (N ​= ​654)	Moderate or severe PPM (N ​= ​125)	sHR_adjusted_ (95% CI)	Adjusted *p*-value	None (N ​= ​366)	Moderate or severe PPM (N ​= ​223)	sHR_adjusted_ (95% CI)	Adjusted *p*-value
At 1 y												
Cardiovascular death, n (%)	59 (5.9)	22 (6.4)	1.15 (0.68-1.93)	0.604	20 (3.1)	4 (3.2)	1.27 (0.41-3.96)	0.678	39 (10.8)	18 (8.2)	0.78 (0.44-1.41)	0.413
Repeat aortic valve intervention, n (%)	8 (0.8)	5 (1.5)	1.72 (0.56-5.30)	0.346	6 (0.9)	1 (0.8)	0.75 (0.09-6.23)	0.789	2 (0.6)	4 (1.8)	2.83 (0.47-17.11)	0.257
Structural valve deterioration, n (%)	23 (2.3)	7 (2.1)	0.88 (0.38-2.05)	0.764	17 (2.7)	2 (1.6)	0.61 (0.14-2.71)	0.514	6 (1.7)	5 (2.3)	1.30 (0.39-4.37)	0.673
At 5 y												
Cardiovascular death, n (%)	151 (31.2)	54 (35.5)	1.24 (0.89-1.72)	0.208	77 (27.6)	13 (29.7)	1.22 (0.66-2.25)	0.523	74 (38.6)	41 (36.5)	1.00 (0.68-1.49)	0.992
Repeat aortic valve intervention, n (%)	10 (1.3)	6 (2.4)	1.71 (0.61-4.76)	0.307	7 (1.3)	1 (1.0)	0.66 (0.08-5.32)	0.698	3 (1.2)	5 (3.4)	2.49 (0.54-11.52)	0.243
Structural valve deterioration, n (%)	26 (3.5)	11 (4.4)	1.24 (0.61-2.53)	0.544	19 (3.9)	4 (4.5)	1.14 (0.38-3.43)	0.818	7 (2.7)	7 (4.5)	1.59 (0.55-4.58)	0.394

*Notes.* Only the first outcome of each outcome type is counted (% from Aalen-Johansson estimates). sHRs from competing risk regression (sHR with 95% CIs) were also adjusted for age, gender, and STS-PROM score (sHR_adjusted_ with 95% CIs). Cardiovascular death is competing with other causes of death; repeat aortic valve intervention and structural valve deterioration are competing with all-cause death. Repeat aortic valve intervention includes valve-in-series, surgical revision, and aortic valve treatment.

Abbreviations: HR, hazard ratio; PPM, ​prosthesis-patient mismatch; PPM_CT_, PPM defined by predicted effective orifice area derived from preprocedural computed tomography; PPM_THV_, PPM defined by predicted effective orifice area for each size and model of implanted transcatheter heart valve; sHR, subdistribution hazard ratio; STS-PROM, ​Society of Thoracic Surgeons Predicted Risk of Mortality.

## Discussion

The main findings of the present study can be summarized as follows: (1) predicted EOAi results in lower estimates of PPM severity compared to measured EOAi in patients undergoing TAVR with a balloon-expandable device; (2) PPM was reclassified in a substantial proportion of patients when flow status-based EOA was considered, especially in patients with low flow after TAVR; (3) there was no consistent signal of increased risk of adverse events over 5 years in patients with flow status-based predicted PPM, regardless of flow status.

Recent studies have revealed a number of limitations of measured EOA. First, echocardiographic measurement results in higher transvalvular gradients and poor agreement with cardiac catheterization due to the pressure recovery phenomenon.^23^^,^^24^ Second, echocardiographic measurements underestimate EOA compared to CT due to the geometric assumption that the cross-sectional area of the left ventricular outflow tract is circular when using 2-dimensional echocardiography.^25^^,^^26^ And third, echocardiography underestimates EOA in patients with low-flow status.^9^ Predicted EOA is the standard reference of EOA for valve type and size or native aortic annulus dimension.^7^ Previous studies have suggested that predicted PPM has a stronger association with hemodynamic outcomes compared to measured PPM and that the use of predicted EOA downgrades the severity of PPM and may more accurately correlate with clinical outcome.^8^, ^9^, ^10^ In a previous analysis, we found a significantly lower 5-year mortality in patients in whom predicted EOA was used to assess PPM as compared to those in whom measured EOA was used. The use of predicted EOA could more accurately predict the true frequency of PPM and more precisely determine the effect of PPM on outcomes.^10^

It should be noted, however, that predicted EOA is limited in its accuracy by failing to account for flow variability. The PARTNER trials, which were the source of the reference values of predicted EOAs, included a non-negligible proportion of patients with low flow status after TAVR (43% in SAPIEN XT and 29% in SAPIEN 3).^11^ The use of a uniform cut-off value of predicted EOA may underestimate or overestimate the severity of PPM in patients with abnormal flow status. In the analysis from the PARTNER trials and registry, the prevalence of moderate or severe predicted PPM_THV_ in patients with low flow status was 18% with SAPIEN XT and 22.9% with SAPIEN 3, respectively, without considering flow variability. The frequency was significantly increased when EOA was adjusted for flow status based on baseline SVI (43% in SAPIEN XT and 45% in SAPIEN 3).^11^ The use of flow status-based EOA could more accurately determine the presence and severity of PPM, especially in patients at a risk for pseudo-PPM. Consistent with the previous study, we found substantial differences in the frequency of predicted and flow status-based predicted PPM in patients with low flow status. In contrast to the study by Akinmolayemi et al., we based the assessment of flow status on SVI after TAVR instead of baseline SVI. As up to 20% of patients with low flow at baseline normalize their flow status and up to 20% of patients with baseline normal flow develop new-onset low flow status,^11^^,^^27^ using flow status-based predicted EOA based on post-TAVR SVI appears to be logical.

Of note, patients with moderate or severe flow status-based predicted PPM_THV_ and PPM_CT_ had a similar 5-year mortality compared to those with no PPM in the present study. The favorable results may be due to the low incidence of severe flow status-based predicted PPM. Previous studies have consistently shown that moderate predicted PPM had no impact on both short- and long-term mortality, and numbers of severe predicted PPM were low.^8^, ^9^, ^10^ Future studies with larger numbers of patients are warranted to confirm our findings and to determine the prognostic impact of flow status-based predicted PPM in TAVR population.

Previous studies have consistently reported that patients who receive a smaller prosthesis have an increased risk of developing PPM after aortic valve replacement.^28^ In the present analysis, patients who developed PPM based on flow status-adjusted predicted EOA were more likely to receive a smaller THV (20 or 23 mm) than those without PPM. Our results suggest that patients with small aortic annuli are still at risk of PPM, even when using more accurate methods to determine EOA in TAVR population. However, it should be noted that the clinical impact of PPM in patients with small annuli is still undetermined. In a recent analysis of pooled data from the PARTNER trials, there were no differences in the incidence of a composite of all-cause death, disabling stroke, or hospitalization for heart failure during 5 years of follow-up between patients with no, moderate, or severe measured PPM in the setting of small aortic annuli defined as aortic annulus area <430 mm^2^.^29^ Future studies are warranted to systematically evaluate the incidence and clinical impact of PPM by each method in patients with small aortic annuli.

Elevated residual prosthetic gradients were more frequent in patients with moderate or severe flow status-based predicted PPM than in those with no PPM. High residual gradients may lead to impaired forward hemodynamics, accelerated bioprosthesis degeneration, and the need for reintervention. However, we observed that the rate of high residual gradients was similar in patients with and without flow status-based predicted PPM in low flow status. These results suggest that residual prosthesis gradients may not be reliable markers to predict patient clinical and bioprosthetic outcomes in patients with low flow status. Further studies, including a larger number of patients with long-term follow-up, are needed to delineate the impact of high residual gradients and PPM after TAVR on clinical outcomes.^1^

Although flow status-based predicted EOA is a more robust parameter to determine the true rate of PPM in TAVR populations, its generalizability needs to be critically evaluated. First, there is a flexible range of device expansion, resulting in various actual EOA even for the same device size and type. Indeed, a prospective post-TAVR CT study has observed that nonuniform expansion and underexpansion were common in patients undergoing TAVR with the SAPIEN 3 prosthesis at different levels of THV.^30^ In addition, predicted EOA_CT_ is based on preprocedural CT and ignores changes in aortic root dimensions after TAVR. A prospective CT study has shown that left ventricular outflow tract area measured by post-TAVR CT was on average 0.30 cm^2^ larger (95% CI, 0.25-0.36; *p* ​< ​ 0.001) compared to pre-TAVR CT and suggested that post-TAVR CT more accurately determines EOA.^26^ Inevitably, SVI may also be more accurately calculated by post-TAVR CT-derived left ventricular outflow tract area. For the current clinical practice, a tailored approach integrating multidisciplinary assessment, including echocardiography, CT, cardiac catheterization, and thorough clinical assessment of residual symptoms after TAVR, is key to the management of patients with suspected PPM by measured and flow status-based predicted EOA. Further research is necessary to establish a standardized assessment method for the evaluation of bioprosthetic valve function.^13^

### Study Limitations

The results of the present study should be interpreted in light of several constraints. First, although this is the first study to investigate the clinical impact of flow status-based predicted PPM, the number of eligible patients was modest. The low prevalence of severe flow status-based predicted PPM and the relatively short median follow-up time warrant cautious interpretation of the results. Similarly, the relatively lower prevalence of female patients may result in a lower frequency of PPM. The impact of PPM may be more pronounced in certain populations.^31^^,^^32^ In turn, we provide comprehensive data on PPM severity using various methods from a large prospective registry that adheres to high standards of data quality with standardized follow-up and independent adjudication of events. Second, since this was a retrospective analysis based on prospectively collected data, the possibility of residual confounding cannot be excluded despite rigorous statistical techniques. Furthermore, post-TAVR SVI in the present study was based on discharge echocardiography, which may change at 30 days. In addition, because EOA is derived from the ratio of SV to aortic valve continuous wave Doppler time velocity integral, a potential interaction between stroke volume index and PPM cannot be ruled out. Third, although the occurrence of structural valve deterioration was systematically recorded and adjudicated, the definitions of structural valve deterioration were not according to the current VARC criteria. Fourth, as the reference value of flow status-based predicted EOA has been proposed only for balloon-expandable devices, the results of the present study cannot be generalized to other types of THV. Fifth, the present cohort included predominantly octogenarians, and the results may not be generalizable to younger patients with fewer comorbidities and longer life expectancy. Finally, although we determined flow status based on SVI in lines with previous studies and current guidelines, SVI related volume and may not perfectly represent aortic valve flow.

## Conclusion

The use of flow status-based as compared to unadjusted predicted EOAi reclassified severity of PPM, particularly in patients with low flow after TAVR. There was no consistent signal of increased risk of adverse events up to 5 years in patients with flow status-based predicted PPM, regardless of flow status. Further studies are warranted to optimize the assessment of bioprosthetic hemodynamics and to evaluate the impact of PPM on long-term clinical outcomes after TAVR.

## Ethics Statement

This study involves human participants and was approved by the Bern Cantonal Ethics Committee (SwissTAVI Kantonale Ethikkommission number 2021-01738). The study was conducted in compliance with the Declaration of Helsinki. Participants gave informed consent to participate in the study before taking part.

## Funding

The authors have no funding to report.

## Data Availability

The data underlying this article will be shared on reasonable request with the corresponding author.

## Disclosure Statement

S. Windecker reports research, travel, or educational grants to the institution without personal remuneration from Abbott, Abiomed, Amgen, Astra Zeneca, Bayer, Braun, Biotronik, Boehringer Ingelheim, Boston Scientific, Bristol Myers Squibb, Cardinal Health, CardioValve, Cordis Medical, Corflow Therapeutics, CSL Behring, Daiichi Sankyo, Edwards Lifesciences, Farapulse Inc, Fumedica, Guerbet, Idorsia, Inari Medical, InfraRedx, Janssen-Cilag, Johnson & Johnson, Medalliance, Medicure, Medtronic, Merck Sharp & Dohm, Miracor Medical, MonarQ, Novartis, Novo Nordisk, Organon, OrPha Suisse, Pharming Tech, Pfizer, Polares, Regeneron, Sanofi-Aventis, Servier, Sinomed, Terumo, Vifor, and V-Wave. S. Windecker served as an advisory board member and/or member of the steering/executive group of trials funded by Abbott, Abiomed, Amgen, Astra Zeneca, Bayer, Boston Scientific, Biotronik, Bristol Myers Squibb, Edwards Lifesciences, MedAlliance, Medtronic, Novartis, Polares, Recardio, Sinomed, Terumo, and V-Wave with payments to the institution but no personal payments. He is also a member of the steering/executive committee group of several investigator-initiated trials that receive funding from industry without impacting his personal remuneration. T. Pilgrim reports research grants to the institution from Edwards Lifesciences, Boston Scientifc and Biotronik, as well as personal fees from Biotronik, Boston Scientific, Metronic, Abbott, and HighLife SAS. D. Reineke reports travel expenses from Abbott, Edwards Lifesciences, and Medtronic. S. Stortecky reports research grants to the institution from Edwards Lifesciences, Medtronic, Boston Scientific, and Abbott and personal fees from Boston Scientific, Teleflex, and BTG. F. Praz reports travel expenses from Abbott, Edwards Lifesciences, and Polares Medical. J. Lanz reports speaker fees to the institution from Edwards Lifesciences and Abbott and served as an advisory board member for Abbott. D. Samim received funding for an online course from Edwards Lifesciences. D. Heg reports and with the Department of Clinical Research, University of Bern, which has a staff policy of not accepting honoraria or consultancy fees. However, Department of Clinical Research is involved in design, conduct, or analysis of clinical studies funded by not-for-profit and for-profit organizations. In particular, pharmaceutical and medical device companies provide direct funding to some of these studies. For an up-to-date list of our conflicts of interest see https://www.ctu.unibe.ch/research_projects/declaration_of_interest/index_eng.html.

The other authors had no conflicts to declare.
