# Supplementary material for: Flow Status-Based Predicted Prosthesis-Patient Mismatch in Patients Undergoing Transcatheter Aortic Valve Replacement With a Balloon-Expandable Valve
Source: Struct Heart. 2024 Dec 6;9(4):100379. doi: 10.1016/j.shj.2024.100379 (PMC12047503; doi:10.1016/j.shj.2024.100379)
Supplement: Supplementary Material [file mmc1.docx]

**SUPPLEMENTAL MATERIAL**

**Flow-Adjusted Predicted Prosthesis–Patient Mismatch in Patients Undergoing Transcatheter Aortic Valve Replacement**

Tomii et al.

**Corresponding Author:**

Thomas Pilgrim, MD, MSc

Department of Cardiology

Inselspital

Bern University Hospital

CH-3010 Bern

Phone: 0041 31 632 21 11

Fax: 0041 31 632 47 70

Mail: [thomas.pilgrim@insel.ch](mailto:thomas.pilgrim@insel.ch)

Table of Contents

[**Supplementary Table 1. Flow-adjusted predicted EOA based on transcatheter heart valve type and size stratified by post-TAVR stroke volume index** 3](#_Toc179786638)

[**Supplementary Table 2. Flow-adjusted predicted EOA based on aortic annulus dimension by pre-procedural computed tomography stratified by post-TAVR stroke volume index** 4](#_Toc179786639)

[**Supplementary Table 3. Baseline and procedural characteristics according to flow-status-based predicted PPM_THV_ in patients with normal or low flow after TAVR** 5](#_Toc179786640)

[**Supplementary Table 4. Baseline and procedural characteristics according to flow-status-based predicted PPM_CT_ in patients with normal or low flow after TAVR** 8](#_Toc179786641)

[**References** 11](#_Toc179786642)

# **Supplementary Table 1. Flow-adjusted predicted EOA based on transcatheter heart valve type and size stratified by post-TAVR stroke volume index**

#

|  | **Flow-adjusted predicted EOA according to Akinmolayemi et al. (1)** | |
| --- | --- | --- |
|  | **Normal flow** | **Low flow** |
| **SAPIEN XT** |  |  |
| 23 mm | 1.5 cm² | 1.3 cm² |
| 26 mm | 1.9 cm² | 1.5 cm² |
| 29 mm | 2.3 cm² | 1.8 cm² |
| **SAPIEN 3 / 3 Ultra** |  |  |
| 20 mm | 1.3 cm² | 1.1 cm² |
| 23 mm | 1.5 cm² | 1.4 cm² |
| 26 mm | 1.8 cm² | 1.6 cm² |
| 29 mm | 2.0 cm² | 1.8 cm² |

EOA: effective orifice area.

# **Supplementary Table 2. Flow-adjusted predicted EOA based on aortic annulus dimension by pre-procedural computed tomography stratified by post-TAVR stroke volume index**

|  | **Flow-adjusted predicted EOA according to Akinmolayemi et al. (1)** | | |
| --- | --- | --- | --- |
|  | **Normal flow** | | **Low flow** |
| **SAPIEN 3 / 3 Ultra** | |  | |
| **Annulus area** | |  | |
| 248 to 384 mm^2^ | 1.4 cm² | | 1.3 cm² |
| 385 to 439 mm^2^ | 1.6 cm² | | 1.5 cm² |
| 440 to 488 mm^2^ | 1.9 cm² | | 1.6 cm² |
| 489 to 537 mm^2^ | 1.9 cm² | | 1.7 cm² |
| 538 to 678 mm^2^ | 2.0 cm² | | 1.8 cm² |

EOA: effective orifice area.

# **Supplementary Table 3. Baseline and procedural characteristics according to flow-status-based predicted PPM_THV_ in patients with normal or low flow after TAVR**

|  | **Flow-status-based predicted PPM_THV_** | | | | | |
| --- | --- | --- | --- | --- | --- | --- |
|  | **Normal flow** | | | **Low flow** | | |
|  | **No PPM**  **(N = 774)** | **Moderate or Severe PPM**  **(N = 83)** | **P value** | **No PPM**  **(N = 407)** | **Moderate or Severe PPM**  **(N = 246)** | **P value** |
| Age, year | 81.4 ± 6.4 | 79.7 ± 6.5 | 0.028 | 81.7 ± 6.7 | 80.9 ± 7.2 | 0.134 |
| Female, n (%) | 284 (36.7%) | 44 (53.0%) | 0.004 | 149 (36.6%) | 85 (34.6%) | 0.614 |
| Body mass index, kg/m^2^ | 26.2 ± 4.8 | 28.4 ± 4.1 | <0.001 | 27.0 ± 5.3 | 28.9 ± 5.7 | <0.001 |
| Body mass index ≥30 kg/m², n (%) | 172 (22.2%) | 11 (13.3%) | 0.066 | 136 (33.4%) | 51 (20.7%) | <0.001 |
| Body surface area, m² | 1.9 ± 0.2 | 2.0 ± 0.2 | <0.001 | 1.9 ± 0.2 | 2.0 ± 0.3 | <0.001 |
| STS PROM, % | 4.5 ± 3.5 | 3.6 ± 2.7 | 0.028 | 5.0 ± 3.9 | 4.8 ± 4.1 | 0.364 |
| NYHA III or IV, n (%) | 417 (53.9%) | 47 (56.6%) | 0.645 | 272 (67.0%) | 158 (64.2%) | 0.496 |
| TAVR for degenerative prosthesis, n (%) | 19 (2.5%) | 3 (3.6%) | 0.464 | 3 (0.7%) | 6 (2.4%) | 0.088 |
| **Concomitant diseases** |  |  |  |  |  |  |
| Hypertension, n (%) | 679 (87.7%) | 78 (94.0%) | 0.105 | 354 (87.0%) | 222 (90.2%) | 0.260 |
| Diabetes mellitus, n (%) | 191 (24.7%) | 28 (33.7%) | 0.085 | 131 (32.2%) | 77 (31.3%) | 0.862 |
| Renal failure (eGFR <60 mL/min/1.73 m^2^), n (%) | 478 (61.8%) | 41 (49.4%) | 0.033 | 262 (64.4%) | 133 (54.1%) | 0.010 |
| Coronary artery disease, n (%) | 463 (59.8%) | 44 (53.0%) | 0.241 | 248 (60.9%) | 141 (57.3%) | 0.367 |
| **Previous history** |  |  |  |  |  |  |
| Atrial fibrillation, n (%) | 213 (27.5%) | 20 (24.1%) | 0.604 | 162 (39.8%) | 98 (39.8%) | 1.00 |
| Peripheral artery disease, n (%) | 91 (11.8%) | 7 (8.4%) | 0.468 | 49 (12.0%) | 37 (15.0%) | 0.284 |
| **Baseline echocardiography** |  |  |  |  |  |  |
| Indexed aortic valve area, cm²/m² | 0.28 ± 0.09 | 0.28 ± 0.08 | 0.800 | 0.28 ± 0.09 | 0.25 ± 0.08 | <0.001 |
| Mean aortic valve gradient, mmHg | 40.9 ± 16.4 | 39.1 ± 13.2 | 0.344 | 36.8 ± 13.8 | 39.1 ± 15.4 | 0.048 |
| Left ventricular ejection fraction, % | 57.2 ± 12.0 | 61.2 ± 8.9 | 0.008 | 51.3 ± 15.2 | 52.4 ± 14.8 | 0.348 |
| Moderate or severe aortic regurgitation, n (%) | 76 (9.8%) | 9 (10.8%) | 0.702 | 24 (5.9%) | 23 (9.4%) | 0.117 |
| Moderate or severe mitral regurgitation, n (%) | 98 (14.7%) | 6 (10.2%) | 0.439 | 74 (21.0%) | 51 (23.8%) | 0.465 |
| Moderate or severe tricuspid regurgitation, n (%) | 50 (8.0%) | 4 (8.0%) | 1.00 | 50 (14.9%) | 31 (16.1%) | 0.708 |
| **Procedural characteristics** |  |  |  |  |  |  |
| General anesthesia, n (%) | 139 (18.0%) | 14 (16.9%) | 0.881 | 65 (16.0%) | 53 (21.5%) | 0.075 |
| Femoral main access site, n (%) | 702 (90.7%) | 78 (94.0%) | 0.420 | 380 (93.4%) | 225 (91.5%) | 0.439 |
| Valve size, mm | 26.2 ± 2.1 | 23.4 ± 1.2 | <0.001 | 26.8 ± 2.0 | 25.3 ± 1.8 | <0.001 |
| Valve size ≤23 mm, n (%) | 154 (19.9%) | 70 (84.3%) | <0.001 | 50 (12.3%) | 80 (32.5%) | <0.001 |
| Pre-dilations, n (%) | 463 (59.9%) | 37 (44.6%) | 0.010 | 216 (53.1%) | 138 (56.3%) | 0.465 |
| Post-dilations, n (%) | 110 (14.2%) | 18 (21.7%) | 0.076 | 39 (9.6%) | 36 (14.6%) | 0.057 |
| Values are mean ± SD or n (%).  eGFR = estimated glomerular filtration rate; NYHA = New York Heart Association; STS‐PROM = society of thoracic surgeons predicted risk of mortality. | | | | | | |

# **Supplementary Table 4. Baseline and procedural characteristics according to flow-status-based predicted PPM_CT_ in patients with normal or low flow after TAVR**

|  | **Flow-status-based predicted PPM_CT_** | | | | | |
| --- | --- | --- | --- | --- | --- | --- |
|  | **Normal flow** | | | **Low flow** | | |
|  | **No PPM**  **(N = 654)** | **Moderate or Severe PPM**  **(N = 125)** | **P value** | **No PPM**  **(N = 366)** | **Moderate or Severe PPM**  **(N = 223)** | **P value** |
| Age, year | 81.4 ± 6.5 | 80.4 ± 5.8 | 0.109 | 81.8 ± 6.5 | 80.5 ± 7.2 | 0.025 |
| Female, n (%) | 240 (36.7%) | 55 (44.0%) | 0.132 | 138 (37.7%) | 75 (33.6%) | 0.332 |
| Body mass index, kg/m^2^ | 26.1 ± 4.8 | 27.8 ± 4.3 | <0.001 | 27.0 ± 5.2 | 29.2 ± 5.9 | <0.001 |
| Body mass index ≥30 kg/m², n (%) | 144 (22.0%) | 17 (13.6%) | 0.040 | 121 (33.1%) | 52 (23.3%) | 0.012 |
| Body surface area, m² | 1.9 ± 0.2 | 2.0 ± 0.2 | <0.001 | 1.9 ± 0.24 | 2.0 ± 0.2 | <0.001 |
| STS PROM, % | 4.4 ± 3.5 | 3.7 ± 2.6 | 0.035 | 4.9 ± 3.7 | 4.5 ± 4.0 | 0.163 |
| NYHA III or IV, n (%) | 363 (55.5%) | 53 (42.4%) | 0.008 | 240 (65.8%) | 142 (63.7%) | 0.656 |
| TAVR for degenerative prosthesis, n (%) | 13 (2.0%) | 3 (2.4%) | 0.731 | 2 (0.5%) | 4 (1.8%) | 0.206 |
| **Concomitant diseases** |  |  |  |  |  |  |
| Hypertension, n (%) | 568 (86.9%) | 119 (95.2%) | 0.006 | 320 (87.4%) | 200 (89.7%) | 0.431 |
| Diabetes mellitus, n (%) | 154 (23.5%) | 36 (28.8%) | 0.213 | 113 (30.9%) | 72 (32.3%) | 0.716 |
| Renal failure (eGFR <60 mL/min/1.73 m^2^), n (%) | 404 (61.9%) | 55 (44.0%) | <0.001 | 226 (61.7%) | 117 (52.5%) | 0.031 |
| Coronary artery disease, n (%) | 388 (59.3%) | 68 (54.4%) | 0.323 | 225 (61.5%) | 130 (58.3%) | 0.488 |
| **Previous history** |  |  |  |  |  |  |
| Atrial fibrillation, n (%) | 178 (27.2%) | 32 (25.6%) | 0.743 | 143 (39.1%) | 86 (38.6%) | 0.931 |
| Peripheral artery disease, n (%) | 76 (11.6%) | 9 (7.2%) | 0.161 | 44 (12.0%) | 34 (15.2%) | 0.263 |
| **Baseline echocardiography** |  |  |  |  |  |  |
| Indexed aortic valve area, cm²/m² | 0.28 ± 0.08 | 0.29 ± 0.09 | 0.418 | 0.27 ± 0.08 | 0.26 ± 0.08 | 0.017 |
| Mean aortic valve gradient, mmHg | 41.3 ± 16.5 | 39.3 ± 14.9 | 0.225 | 37.0 ± 14.0 | 39.6 ± 15.2 | 0.036 |
| Left ventricular ejection fraction, % | 57.0 ± 12.4 | 61.1 ± 7.5 | 0.001 | 50.8 ± 15.5 | 54.6 ± 13.2 | 0.003 |
| Moderate or severe aortic regurgitation, n (%) | 61 (9.3%) | 13 (10.4%) | 0.739 | 25 (6.8%) | 20 (9.0%) | 0.341 |
| Moderate or severe mitral regurgitation, n (%) | 80 (14.3%) | 7 (7.1%) | 0.053 | 67 (21.5%) | 37 (18.6%) | 0.499 |
| Moderate or severe tricuspid regurgitation, n (%) | 40 (7.6%) | 7 (7.9%) | 1.00 | 42 (14.4%) | 27 (14.8%) | 1.00 |
| **Procedural characteristics** |  |  |  |  |  |  |
| General anesthesia, n (%) | 113 (17.3%) | 18 (14.4%) | 0.514 | 56 (15.3%) | 43 (19.3%) | 0.214 |
| Femoral main access site, n (%) | 601 (91.9%) | 117 (93.6%) | 0.590 | 342 (93.4%) | 207 (92.8%) | 0.866 |
| Valve size, mm | 26.3 ± 2.1 | 24.2 ± 1.6 | <0.001 | 26.8 ± 2.1 | 25.4 ± 1.9 | <0.001 |
| Valve size ≤23 mm, n (%) | 124 (19.0%) | 80 (64.0%) | <0.001 | 50 (13.7%) | 67 (30.0%) | <0.001 |
| Pre-dilations, n (%) | 380 (58.1%) | 55 (44.0%) | 0.004 | 192 (52.5%) | 117 (52.5%) | 1.00 |
| Post-dilations, n (%) | 91 (13.9%) | 24 (19.2%) | 0.131 | 44 (12.0%) | 28 (12.6%) | 0.897 |
| Values are mean ± SD or n (%).  eGFR = estimated glomerular filtration rate; NYHA = New York Heart Association; STS‐PROM = society of thoracic surgeons predicted risk of mortality. | | | | | | |

## **References**

1. Akinmolayemi O, Ozdemir D, Pibarot P, et al. Clinical and Echocardiographic Characteristics of Flow-Based Classification Following Balloon-Expandable Transcatheter Heart Valve in PARTNER Trials. JACC Cardiovasc Imaging 2023;16:1-9.
